# Sex differences in mortality: results from a population-based study of 12 longitudinal cohorts

**DOI:** 10.1503/cmaj.200484

**Published:** 2021-03-15

**Authors:** Yu-Tzu Wu, Albert Sanchez Niubo, Christina Daskalopoulou, Dario Moreno-Agostino, Denes Stefler, Martin Bobak, Sian Oram, Martin Prince, Matthew Prina

**Affiliations:** Department of Health Service and Population Research (Wu, Daskalopoulou, Moreno-Agostino, Oram, Prince, Prina), Institute of Psychiatry, Psychology and Neuroscience, King’s College London, London, UK; Population Health Sciences (Wu), Newcastle University, Newcastle upon Tyne, UK; Research, Innovation and Teaching Unit (Sanchez Niubo), Parc Sanitari Sant Joan de Déu, Sant Boi de Llobregat, Spain; Centro de Investigación Biomédica en Red de Salud Mental (Sanchez Niubo), Madrid, Spain; Department of Epidemiology and Public Health (Stefler, Bobak), University College London, London, UK

## Abstract

**BACKGROUND:**

Women generally have longer life expectancy than men but have higher levels of disability and morbidity. Few studies have identified factors that explain higher mortality in men. The aim of this study was to identify potential factors contributing to sex differences in mortality at older age and to investigate variation across countries.

**METHODS:**

This study included participants age ≥ 50 yr from 28 countries in 12 cohort studies of the Ageing Trajectories of Health: Longitudinal Opportunities and Synergies (ATHLOS) consortium. Using a 2-step individual participant data meta-analysis framework, we applied Cox proportional hazards modelling to investigate the association between sex and mortality across different countries. We included socioeconomic (education, wealth), lifestyle (smoking, alcohol consumption), social (marital status, living alone) and health factors (cardiovascular disease, diabetes, mental disorders) as covariates or interaction terms with sex to test whether these factors contributed to the mortality gap between men and women.

**RESULTS:**

The study included 179 044 individuals. Men had 60% higher mortality risk than women after adjustment for age (pooled hazard ratio [HR] 1.6; 95% confidence interval 1.5–1.7), yet the effect sizes varied across countries (*I*^2^ = 71.5%, HR range 1.1–2.4). Only smoking and cardiovascular diseases substantially attenuated the effect size (by about 22%).

**INTERPRETATION:**

Lifestyle and health factors may partially account for excess mortality in men compared with women, but residual variation remains unaccounted for. Variation in the effect sizes across countries may indicate contextual factors contributing to gender inequality in specific settings.

Life expectancy has increased over the last 6 decades in many societies around the world.^1^ Women generally have longer life expectancy than men, yet have higher levels of disability and morbidity.^2^,^3^ Male:female mortality ratios increased from the beginning of the 19th century and slightly decreased over the last 3 decades.^4^,^5^ It has been suggested that the biological differences between the sexes, including genetics and hormones, provide stronger resilience to disadvantageous situations for women than men.^6^ However, biological sex is related to gender, a construct that also incorporates cultural and social differences between men and women. Although some studies suggest that the recent reduction in the male:female mortality ratio is likely a result of improvements in men’s health, lifestyle or occupational environments, others attribute it to women’s changing societal roles and increasing mortality from diseases such as lung cancer, which have traditionally affected mostly men.^3^,^7^–^9^ Many studies have examined the potential impact of social, behavioural and biological factors on sex differences in mortality,^10^,^11^ but few have been able to investigate potential variation across countries. Different cultural traditions, historical contexts, and economic and societal development may influence gender experiences in different countries, and thus variably affect the health status of men and women.

We aimed to identify factors that may explain the difference in mortality risk between men and women at older age and to investigate potential variation across countries, using the harmonized data set of 12 cohort studies from the Ageing Trajectories of Health: Longitudinal Opportunities and Synergies (ATHLOS) consortium.^12^

## Methods

### Study population

The ATHLOS project is a consortium funded by the European Union’s Horizon 2020 research and innovation program (grant agreement no. 635316).^12^ The project aims to identify healthy aging trajectories and their determinants, using existing aging cohorts around the world. Most of the ATHLOS cohorts were established after 2000. Researchers in the consortium reviewed information from 17 cohort studies and agreed on approaches to harmonize measures for lifestyle, social environment, physical and psychological health across cohorts.

This study focused on participants aged 50 years or older in the 12 cohort studies with available mortality data, including the 10/66 Dementia Research Group Study (the 10/66 study);^13^ the Australian Longitudinal Study of Ageing (ALSA);^14^ the ATTICA study;^15^ the China Health and Retirement Longitudinal Study (CHARLS);^16^ the Collaborative Research on Ageing in Europe (COURAGE);^17^ the English Longitudinal Study of Ageing (ELSA);^18^ the Seniors-ENRICA (the Study on Nutrition and Cardiovascular Risk in Spain) study;^19^ the Health, Alcohol and Psychosocial factors In Eastern Europe (HAPIEE) study;^20^ the Health and Retirement Study (HRS);^21^ the Japanese Study of Aging and Retirement (JSTAR);^22^ the Korean Longitudinal Study of Aging (KLOSA);^23^ and the Survey of Health, Ageing and Retirement in Europe (SHARE).^24^ These 12 cohort studies had recruited community-dwelling older adults from 28 countries (Table 1) and used structured interviews to collect individual data. More detailed information on the study population is provided in Appendix 1, Table S1 (available at www.cmaj.ca/lookup/doi/10.1503/cmaj.200484/tab-related-content).

**Table 1:** Descriptive information on the study population of community-dwelling older adults, by country

Country	*n*	Age	Sex	Socioeconomic*		Lifestyle*	
			
		Median (IQR)	Women, %	Low education, %	Low wealth, %	Current smoker, %	Alcohol consumption: never, %
Cuba	2801	74 (11)	65.3	57.9	27.9	19.4	87.5

Dominican Republic	2009	74 (11)	66.0	89.6	35.0	12.4	82.2

Mexico	2002	73 (10)	63.3	88.4	31.4	9.0	81.3

Peru	1933	74 (11)	61.2	56.3	35.4	3.5	95.1

Puerto Rico	2002	76 (11)	67.3	43.8	20.1	5.2	89.0

United States	35 747	57 (17)	55.6	27.2	20.3	21.4	52.3

Venezuela	1958	71 (10)	63.7	81.3	32.0	11.5	76.5

China	15 924	62 (14)	51.2	72.6	24.6	28.9	70.7

Israel	2545	64 (15)	55.1	24.0	21.4	15.4	71.1

Japan	5144	63 (12)	50.9	29.6	25.2	22.1	44.2

South Korea	8466	64 (15)	56.5	53.0	23.9	18.4	10.0

Austria	4769	64 (14)	57.4	14.0	19.5	19.6	27.0

Belgium	5413	61 (17)	54.1	21.2	19.6	19.3	19.6

Czech Republic	12 359	61 (11)	54.8	15.0	19.1	23.7	23.4

Denmark	2982	60 (16)	53.4	14.1	16.4	29.1	8.0

Estonia	5712	66 (16)	59.9	6.2	20.0	19.7	40.8

France	5700	62 (17)	55.7	40.0	20.8	15.3	22.6

Germany	2479	63 (14)	52.5	0.8	18.0	18.0	21.5

Greece	4371	60 (16)	52.8	49.6	21.6	29.3	44.0

Italy	3833	63 (14)	54.1	51.4	19.2	19.0	44.6

Netherlands	3532	60 (14)	53.5	13.7	17.5	22.7	22.7

Poland	10 842	60 (10)	51.3	18.8	19.1	29.1	38.8

Slovenia	1974	64 (16)	56.5	14.7	19.5	14.3	45.9

Spain	10 377	66 (15)	54.2	61.7	20.3	16.8	40.6

Sweden	3220	64 (15)	53.3	35.0	20.0	17.1	18.4

Switzerland	3367	63 (16)	53.4	10.8	18.0	19.5	14.1

UK	15 518	61 (16)	54.1	38.7	18.5	17.6	11.3

Australia	2065	78 (10)	48.9	36.5	36.7	8.1	37.2

Missing		–	–	1.8	14.2	1.9	3.9

Total	179 044	63 (16)	55.0	36.3	21.6	20.7	41.6

Country	*n*	Age	Sex	Health				Social	
			
		Median (IQR)	Women, %	CVD, %	Diabetes, %	Hypertension, %	Depression, %	No spouse, %	Living alone, %
Cuba	2801	74 (11)	65.3	35.2	18.6	51.5	16.8	57.3	8.7

Dominican Republic	2009	74 (11)	66.0	18.6	14.1	61.8	26.9	70.6	12.6

Mexico	2002	73 (10)	63.3	18.2	21.7	46.8	17.9	49.7	10.8

Peru	1933	74 (11)	61.2	21.1	9.0	46.2	19.2	43.2	4.6

Puerto Rico	2002	76 (11)	67.3	29.7	32.1	69.3	10.6	51.7	23.5

United States	35 747	57 (17)	55.6	18.4	12.5	39.8	16.0	29.5	17.5

Venezuela	1958	71 (10)	63.7	25.5	16.2	62.5	19.7	52.2	3.1

China	15 924	62 (14)	51.2	16.6	7.0	29.1	33.7	17.8	11.5

Israel	2545	64 (15)	55.1	21.8	21.8	44.0	35.9	21.2	14.3

Japan	5144	63 (12)	50.9	15.6	12.1	36.2	17.4	20.4	-

South Korea	8466	64 (15)	56.5	9.4	13.6	31.4	34.8	24.9	10.0

Austria	4769	64 (14)	57.4	14.7	13.6	36.5	20.4	34.5	29.9

Belgium	5413	61 (17)	54.1	15.4	10.1	31.7	28.0	27.9	22.4

Czech Republic	12 359	61 (11)	54.8	17.3	17.5	59.4	21.1	27.2	23.1

Denmark	2982	60 (16)	53.4	13.0	8.4	29.6	17.5	29.2	23.6

Estonia	5712	66 (16)	59.9	28.2	16.2	49.1	41.0	35.3	24.1

France	5700	62 (17)	55.7	15.0	11.9	29.4	33.2	31.4	24.6

Germany	2479	63 (14)	52.5	14.5	12.7	36.4	18.5	21.3	15.9

Greece	4371	60 (16)	52.8	17.7	13.0	38.0	23.4	25.0	22.9

Italy	3833	63 (14)	54.1	13.2	13.6	38.0	33.0	17.5	10.0

Netherlands	3532	60 (14)	53.5	13.6	9.2	26.0	19.0	17.9	15.7

Poland	10 842	60 (10)	51.3	25.5	14.1	60.5	30.5	23.5	10.2

Slovenia	1974	64 (16)	56.5	17.0	15.7	42.3	26.8	24.6	19.1

Spain	10 377	66 (15)	54.2	10.7	17.6	44.2	22.4	29.3	15.6

Sweden	3220	64 (15)	53.3	18.7	9.4	30.0	18.9	21.9	20.5

Switzerland	3367	63 (16)	53.4	9.6	7.9	28.7	18.2	25.0	21.3

UK	15 518	61 (16)	54.1	15.6	7.1	34.5	16.6	27.5	21.2

Australia	2065	78 (10)	48.9	15.9	8.8	33.0	15.4	35.4	26.6

Missing		–	–	1.2	2.6	1.5	3.7	0.1	12.6

Total	179 044	63 (16)	55.0	17.4	12.7	40.4	23.4	28.3	17.5

Note: CVD = cardiovascular disease, IQR = interquartile range.

* Categories of socioeconomic and lifestyle factors: Education: low (up to primary education), middle (secondary) and high levels (tertiary); Wealth: first (low) to fifth quintile (high); Smoking: current, ever, never; Alcohol consumption: never, rare (once or less a week) and often (twice or more per week).

### Outcomes and variables

We obtained all data from the ATHLOS harmonized data set, which provides comparable measures across cohort studies. The primary outcome was mortality. Information on date of death was collected using death registers (ALSA, COURAGE, ELSA, HAPIEE, Seniors-ENRICA, HRS, JSTAR, KLOSA, SHARE) or interviews with participants’ family or informants (the 10/66 study, CHARLS, ATTICA). The censoring time was set to be the end of follow-up in each cohort study. The longest follow-up period was 20 years in ALSA and HRS, and the shortest was 2 years in CHARLS.

Other variables of interest included socioeconomic (education, wealth), lifestyle (smoking, alcohol consumption), health (cardiovascular disease, diabetes, hypertension, depression) and social factors (living alone, no spouse or partner) at baseline. These variables were selected as most cohorts had available data. We categorized education into low (up to primary education), middle (secondary) and high (tertiary) levels. Wealth was based on individual or household income and other financial information (such as pensions, insurance) and was divided into quintiles within specific cohorts. Self-reported smoking status included 3 groups: nonsmoker, ex-smoker and current smoker. Alcohol consumption was based on self-reported frequency and categorized into 3 groups: never, rare (≤ 1 time/wk) and often (≥ 2 times/wk). Four types of health conditions (cardiovascular disease, diabetes, hypertension and depression) were recorded as binary variables (yes or no). We used self-reported diagnosis of cardiovascular disease (any of angina, stroke, myocardial infarction, heart attack, coronary heart disease, congestive heart failure, heart murmur, valve disease and cerebral vascular disease), diabetes and hypertension to identify patients with these conditions. We determined depression status using the available assessment tools and their established cut-offs in specific cohort studies. We dichotomized (yes or no) 2 social factors, living alone and no spouse or partner, based on self-reported information. The Japanese cohort (JSTAR) did not have data on living alone; therefore, analyses related to this variable included only 27 countries.

To establish baseline conditions of these factors, we obtained all variables from the first wave if available. If some variables were not available at the baseline, we used information from the second or third follow-up waves to inform possible conditions of participants. More details on data harmonization are available online at https://github.com/athlosproject/athlos-project.github.io.

To contextualize the results, we obtained the gender inequality index and the gender development index for each country from the United Nations (UN) Human Development Reports 2018.^25^ The gender inequality index focuses on the disadvantages that women face in terms of reproductive health, empowerment and labour market.^26^ The gender development index was developed to measure disparities of human development achievements between men and women, including health, knowledge and standard of living.^26^

### Statistical analysis

A factor might lead to sex differences in mortality in 2 ways. First, if a factor has similar effects on females and males, the variation could be a result of differential prevalence of this factor across the sexes. Higher mortality in males could be (largely or partially) attributed to higher prevalence of this factor. Adjusting for this factor as a covariate should attenuate the effect size of sex. Second, if a factor has different effects on females and males, sex differences might not be attenuated by the prevalence of this factor. In this case, the model should include the interaction terms between sex and this factor to account for its effect. To estimate hazard ratios of mortality between men and women, we carried out Cox proportion hazards modelling to include different factors as covariates (Model *a*) or their interaction terms with sex (Model *b*). We fitted each factor individually in Model *a* or Model *b* to investigate changes in the sex coefficient compared with the original model, which included only sex and age. We included factors that were identified to attenuate age-adjusted hazard ratios between men and women in 1 multivariable model. We estimated the marginal effects of sex on mortality for Model *a* and *b* with adjustment for age and calculated the percentage of change in estimates. According to the literature, a 10% change between unadjusted and adjusted estimates is generally used in confounder selection.^27^ Given that multiple factors could be related to sex differences in mortality, we used a > 5% attenuation to indicate partial contribution of a factor.

We used a 2-stage individual participant–data analytical approach^28^ to generate country-specific and overall pooled estimates. Given the large heterogeneity, we used a random-effects meta-analysis model to identify the distribution of sex differences in mortality across countries. We used Spearman rank correlation to estimate the direction and strength of monotonic relationships between adjusted hazard ratios and the gender inequality and gender development indicators individually.

We examined the proportional hazards assumption using the Schoenfeld residual test and interaction terms between time and covariates. Because the effect sizes were generally small, modelling did not further include variables with time-varying effects (Appendix 1, Table S2). We carried out full models including all socioeconomic, lifestyle, health and social factors (with or without living alone) as sensitivity analyses (Appendix 1, Table S3). To address time-varying covariates, we performed additional sensitivity analyses to incorporate lifestyle, health and social factors at different waves in modelling (Appendix 1, Table S4). To test the impact of birth cohorts, sensitivity analyses further included birth cohorts and their interaction terms with age in the modelling (Appendix 1, Table S5). We carried out all analyses using Stata 15.0.

### Ethics approval

The project was approved by the research ethics committee at Fundación Sant Joan de Déu, Barcelona, Spain (code 635316-2).

## Results

We included 179 044 participants in the analysis. The median age of participants was 63 years, with an interquartile range (IQR) between 55 and 71 years (Table 1). Nearly 55% were women (*n* = 98 430). The median period of follow-up was 4 years (IQR 5 yr), and 14.7% (*n* = 26 484) died at the end of follow-up. Among the participants, 36.3% had primary education or less, 20.7% were current smokers and just more than 40% abstained from alcohol. Among the 4 health conditions, hypertension (40.4%) was the most frequent and the least frequent was diabetes (12.7%). There were 28.3% participants without a spouse and 17.5% lived alone. More detailed results, stratified by sex, are reported in Appendix 1, Table S6.

**Figure 1:**
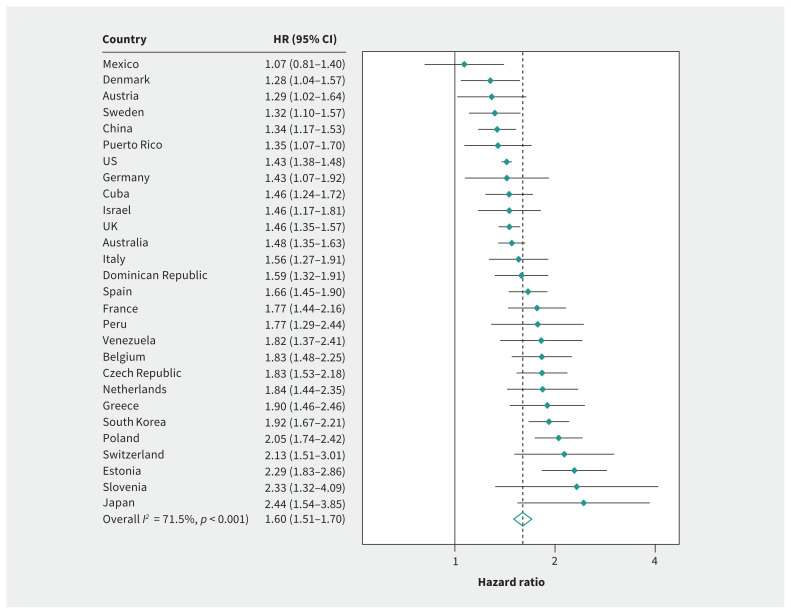
Age-adjusted hazard ratios of mortality between men and women (reference). Note: CI = confidence interval, HR = hazard ratio.

Based on the pooled estimate, men had a 60% higher mortality risk than women (1.60; 95% confidence interval [CI] 1.51–1.70). The heterogeneity (*I*^2^) across countries was 71.5%, with a range from 1.07 (95% CI 0.81–1.41) in Mexico to 2.44 (95% CI 1.54–3.85) in Japan (Figure 1). The strength of association between sex and mortality did not decrease when we adjusted for most socioeconomic, lifestyle, social or health factors (Table 2). Sex differences in mortality became wider when we accounted for education, wealth, alcohol consumption, depression and no spouse. Only adjustment for smoking (1.47; 95% CI 1.39–1.55) and cardiovascular disease (1.56; 95% CI 1.46–1.66) slightly attenuated sex differences in mortality. Country-specific estimates are reported in Appendix 1, Table S7. The amount of reduction was similar across Model *a* and Model *b* (Appendix 1, Table S8). Only a small number of interaction terms with sex achieved statistical significance.

**Table 2:** Pooled estimates for age-adjusted hazard ratios of mortality between men and women (reference) adjusted for socioeconomic, lifestyle, health and social factors

Variable	Pooled HR (95% CI)	*I*^2^, %	Change, %*
Age	1.60 (1.51–1.70)	71.5	–
Socioeconomic			
Education	1.68 (1.58–1.80)	76.1	10.4
Wealth	1.64 (1.56–1.73)	52.5	5.3
Lifestyle			
Smoking	1.47 (1.39–1.55)	57.6	−18.0
Alcohol consumption	1.74 (1.62–1.88)	79.3	17.8
Health			
Cardiovascular disease	1.56 (1.46–1.66)	73.6	−5.4
Diabetes	1.58 (1.49–1.68)	67.0	−2.7
Hypertension	1.61 (1.52–1.70)	69.1	1.3
Depression	1.72 (1.61–1.85)	77.7	15.4
Social			
No spouse	1.71 (1.60–1.82)	72.2	14.1
Living alone	1.57 (1.49–1.65)	53.1	−4.0

Note: CI = confidence interval, HR = hazard ratio.

* Change, %: the percentage change in effect size compared with the age-adjusted HR on the logarithmic scale.

Because most attenuations were found in smoking and cardiovascular disease (Appendix 1, Table S8), we stratified age-adjusted hazard ratios by smoking (Table 3), and by smoking and cardiovascular disease (Table 4). The overall sex differences reduced in nonsmokers (1.40; 95% CI 1.32–1.49) and ex-smokers (1.43; 95% CI 1.32–1.56). Adjustment for smoking and cardiovascular disease further attenuated the hazard ratio slightly (1.44; 95% CI 1.36–1.52). When stratified by smoking status and cardiovascular disease, the overall sex differences decreased to 1.34 (95% CI 1.25–1.44) in nonsmokers who did not have cardiovascular disease.

**Table 3:** Age-adjusted hazard ratios of mortality between men and women (reference), adjusted for smoking

Country	Model 1: adjusted for age	Model 2*a*: adjusted for age, smoking		Model 2 *b*: adjusted for age, interaction between sex and smoking status			
	
			Change, %*	Nonsmoker	Ex-smoker	Current smoker	Change, %*
Cuba	1.5 (1.2–1.7)	1.3 (1.1–1.6)	−24.2	1.4 (1.1–1.9)	1.1 (0.8–1.6)	1.5 (1.0–2.1)	−22.3

Dominican Republic	1.6 (1.3–1.9)	1.5 (1.3–1.9)	−6.0	1.3 (1.0–1.8)	1.9 (1.4–2.6)	1.3 (0.8–2.1)	−10.4

Mexico	1.1 (0.8–1.4)	1.1 (0.7–1.5)	−19.6	1.3 (0.9–2.0)	0.6 (0.3–1.0)	0.9 (0.2–4.2)	−17.7

Peru	1.8 (1.3–2.4)	1.7 (1.2–2.4)	−6.7	1.7 (1.2–2.5)	1.4 (0.5–3.7)	1.8 (0.2–16.0)	−7.6

Puerto Rico	1.4 (1.1–1.7)	1.2 (0.9–1.5)	−46.7	1.3 (1.0–1.8)	1.0 (0.6–1.6)	0.9 (0.4–2.0)	−34.9

United States	1.4 (1.4–1.5)	1.3 (1.3–1.4)	−24.9	1.2 (1.2–1.3)	1.3 (1.2–1.4)	1.4 (1.3–1.5)	−27.3

Venezuela	1.8 (1.4–2.4)	1.9 (1.4–2.5)	3.0	2.0 (1.3–3.1)	1.5 (0.9–2.5)	2.3 (0.9–5.8)	5.5

China	1.3 (1.2–1.5)	1.2 (1.0–1.4)	−42.0	1.2 (1.0–1.4)	1.3 (0.8–2.3)	1.1 (0.8–1.5)	−43.8

Israel	1.5 (1.2–1.8)	1.5 (1.2–1.8)	−0.8	1.3 (1.0–1.8)	1.2 (0.7–1.8)	3.4 (1.7–6.8)	1.5

Japan	2.4 (1.5–3.9)	1.8 (1.0–3.2)	−33.5	2.4 (1.1–4.9)	0.7 (0.3–1.8)	2.9 (0.7–12.1)	−33.1

South Korea	1.9 (1.7–2.2)	1.7 (1.4–2.0)	−22.7	1.7 (1.4–2.1)	2.4 (1.0–5.9)	1.2 (0.8–1.8)	−20.7

Austria	1.3 (1.0–1.6)	1.3 (1.0–1.7)	−1.4	1.3 (1.0–1.8)	1.3 (0.7–2.4)	1.1 (0.6–2.0)	−4.2

Belgium	1.8 (1.5–2.2)	1.7 (1.3–2.1)	−15.5	1.7 (1.2–2.4)	1.8 (1.1–3.0)	1.5 (0.9–2.5)	−14.1

Czech Republic	1.8 (1.5–2.2)	1.7 (1.4–2.0)	−14.6	1.4 (1.1–1.8)	2.0 (1.3–3.1)	2.3 (1.5–3.5)	−8.8

Denmark	1.3 (1.0–1.6)	1.2 (1.0–1.5)	−16.0	1.2 (0.8–1.8)	1.3 (0.9–1.9)	1.2 (0.8–1.6)	−14.5

Estonia	2.3 (1.8–2.9)	1.6 (1.3–2.1)	−40.5	1.3 (0.9–1.9)	1.7 (1.0–2.8)	2.8 (1.4–5.7)	−41.7

France	1.8 (1.4–2.2)	1.6 (1.2–2.0)	−14.4	1.5 (1.1–2.0)	1.9 (1.2–3.0)	2.0 (1.0–3.8)	−11.7

Germany	1.4 (1.1–1.9)	1.3 (0.9–1.8)	−29.6	1.3 (0.8–1.9)	1.3 (0.6–2.5)	1.4 (0.7–2.8)	−31.0

Greece	1.9 (1.5–2.5)	1.8 (1.3–2.4)	−11.1	1.5 (1.1–2.1)	1.9 (0.8–4.8)	4.2 (1.6–10.8)	16.8

Italy	1.6 (1.3–1.9)	1.4 (1.1–1.7)	−27.1	1.2 (0.9–1.6)	1.4 (0.9–2.3)	2.4 (1.3–4.3)	−23.1

Netherlands	1.8 (1.4–2.3)	1.9 (1.4–2.4)	1.2	2.3 (1.5–3.5)	1.7 (1.0–2.7)	1.5 (0.9–2.6)	1.1

Poland	2.1 (1.7–2.4)	1.7 (1.4–2.0)	−27.2	1.1 (0.8–1.5)	2.1 (1.5–3.0)	2.2 (1.6–3.0)	−32.0

Slovenia	2.3 (1.3–4.1)	2.0 (1.1–3.7)	−17.9	2.0 (1.0–4.2)	1.1 (0.3–4.2)	4.7 (0.6–38.4)	−17.3

Spain	1.7 (1.5–1.9)	1.7 (1.4–2.0)	0.4	1.5 (1.3–1.9)	2.1 (1.3–3.4)	2.2 (1.1–4.2)	13.1

Sweden	1.3 (1.1–1.6)	1.3 (1.0–1.5)	−18.0	1.1 (0.9–1.5)	1.2 (0.9–1.7)	1.8 (1.1–2.8)	−16.9

Switzerland	2.1 (1.5–3.0)	2.2 (1.5–3.2)	4.2	2.1 (1.3–3.5)	1.9 (0.8–4.4)	2.9 (1.2–6.7)	2.4

United Kingdom	1.5 (1.4–1.6)	1.4 (1.3–1.5)	−6.3	1.5 (1.3–1.7)	1.4 (1.2–1.5)	1.5 (1.3–1.8)	−4.9

Australia	1.5 (1.4–1.6)	1.4 (1.2–1.5)	−17.0	1.4 (1.2–1.6)	1.5 (1.2–1.7)	1.3 (0.9–1.8)	−15.7

Pooled estimate	1.6 (1.5–1.7)	1.5 (1.4–1.5)		1.4 (1.3–1.5)	1.4 (1.3–1.6)	1.6 (1.4–1.8)	

*I*^2^	71.5	57.6		35.7	33.2	43.8	

* Change (%): the percentage change in effect size compared with the age-adjusted hazard ratio on the logarithmic scale.

**Table 4:** Age-adjusted hazard ratios of mortality between men and women (reference), adjusting for smoking and cardiovascular disease

Country	Model 1: adjusted for age	Model *a*: adjusted for age, smoking, CVD	Model *b*: adjusted for age, interaction between sex, smoking and CVD			
			No CVD, never smoker	No CVD, ever smoker	CVD, never smoker	CVD, ever smoker
Cuba	1.5 (1.2–1.7)	1.3 (1.1–1.6)	1.5 (1.0–2.1)	1.5 (1.1–2.2)	1.4 (0.9–2.1)	1.0 (0.7–1.4)
Dominican Republic	1.6 (1.3–1.9)	1.6 (1.3–1.9)	1.3 (0.9–1.8)	1.7 (1.2–2.2)	1.7 (0.9–3.0)	1.9 (1.1–3.2)
Mexico	1.1 (0.8–1.4)	1.0 (0.7–1.5)	1.3 (0.8–2.0)	0.7 (0.4–1.5)	1.2 (0.5–2.8)	0.2 (0.1–0.7)
Peru	1.8 (1.3–2.4)	1.8 (1.3–2.5)	1.8 (1.2–2.8)	1.7 (0.5–5.8)	2.1 (1.1–4.0)	1.3 (0.3–4.7)
Puerto Rico	1.4 (1.1–1.7)	1.2 (0.9–1.5)	1.4 (0.9–2.1)	0.8 (0.5–1.4)	1.3 (0.8–2.1)	1.1 (0.6–2.1)
United States	1.4 (1.4–1.5)	1.3 (1.2–1.3)	1.2 (1.1–1.3)	1.3 (1.2–1.4)	1.2 (1.0–1.3)	1.1 (1.0–1.2)
Venezuela	1.8 (1.4–2.4)	1.9 (1.4–2.6)	1.8 (1.1–3.0)	1.1 (0.6–2.0)	3.7 (1.7–8.4)	2.6 (1.3–5.2)
China	1.3 (1.2–1.5)	1.2 (1.0–1.4)	1.1 (0.9–1.3)	1.3 (0.9–1.8)	1.5 (1.1–2.1)	0.9 (0.6–1.5)
Israel	1.5 (1.2–1.8)	1.3 (1.1–1.7)	1.3 (0.9–1.9)	1.7 (1.1–2.8)	1.1 (0.7–1.8)	1.1 (0.6–1.9)
Japan	2.4 (1.5–3.9)	2.0 (1.1–3.6)	3.0 (1.2–7.6)	2.1 (0.6–6.8)	1.8 (0.5–6.3)	0.6 (0.2–2.2)
South Korea	1.9 (1.7–2.2)	1.6 (1.4–1.9)	1.6 (1.3–1.9)	1.4 (0.9–2.0)	2.0 (1.4–3.0)	1.5 (0.6–3.8)
Austria	1.3 (1.0–1.6)	1.2 (1.0–1.6)	1.4 (1.0–2.0)	1.0 (0.6–1.7)	1.2 (0.6–2.3)	0.9 (0.4–1.9)
Belgium	1.8 (1.5–2.2)	1.6 (1.3–2.1)	1.3 (0.9–2.1)	1.3 (0.9–2.1)	2.1 (1.2–3.9)	1.9 (1.0–3.5)
Czech Republic	1.8 (1.5–2.2)	1.6 (1.3–1.9)	1.3 (0.9–1.8)	2.0 (1.4–2.9)	1.4 (1.0–2.1)	2.0 (1.1–3.7)
Denmark	1.3 (1.0–1.6)	1.2 (1.0–1.5)	0.9 (0.6–1.5)	1.0 (0.8–1.4)	2.4 (1.1–5.2)	1.5 (1.0–2.4)
Estonia	2.3 (1.8–2.9)	1.6 (1.2–2.1)	1.3 (0.7–2.3)	2.1 (1.2–3.5)	1.2 (0.7–2.2)	2.0 (1.0–3.7)
France	1.8 (1.4–2.2)	1.5 (1.2–2.0)	1.1 (0.7–1.6)	1.9 (1.2–2.9)	2.0 (1.3–3.2)	1.5 (0.8–3.1)
Germany	1.4 (1.1–1.9)	1.3 (0.9–1.7)	1.1 (0.7–1.9)	1.1 (0.6–1.9)	1.2 (0.6–2.4)	1.5 (0.6–4.0)
Greece	1.9 (1.5–2.5)	1.7 (1.2–2.3)	2.0 (1.3–3.1)	2.9 (1.2–6.8)	0.9 (0.5–1.6)	2.8 (0.9–9.1)
Italy	1.6 (1.3–1.9)	1.3 (1.1–1.7)	1.4 (1.0–2.0)	1.6 (1.0–2.5)	0.5 (0.2–1.0)	1.6 (0.8–3.1)
Netherlands	1.8 (1.4–2.3)	1.8 (1.4–2.4)	2.3 (1.4–3.7)	1.3 (0.9–1.9)	2.1 (0.9–4.7)	1.9 (0.9–4.0)
Poland	2.1 (1.7–2.4)	1.7 (1.4–2.0)	1.0 (0.7–1.4)	2.0 (1.5–2.7)	1.3 (0.8–2.3)	2.2 (1.4–3.4)
Slovenia	2.3 (1.3–4.1)	2.0 (1.1–3.6)	1.9 (0.8–4.7)	3.3 (0.8–14.6)	2.1 (0.6–7.1)	0.4 (0.1–2.3)
Spain	1.7 (1.5–1.9)	1.7 (1.4–2.0)	1.4 (1.1–1.8)	2.3 (1.4–3.6)	2.2 (1.4–3.3)	1.5 (0.7–3.2)
Sweden	1.3 (1.1–1.6)	1.2 (1.0–1.4)	1.2 (0.9–1.8)	1.3 (0.9–1.7)	0.9 (0.6–1.4)	1.0 (0.6–1.7)
Switzerland	2.1 (1.5–3.0)	2.2 (1.5–3.2)	2.0 (1.2–3.5)	1.7 (0.9–3.2)	2.1 (0.6–7.4)	6.4 (0.9–48.5)
United Kingdom	1.5 (1.4–1.6)	1.4 (1.3–1.5)	1.4 (1.2–1.7)	1.3 (1.2–1.5)	1.4 (1.0–1.8)	1.3 (1.1–1.5)
Australia	1.5 (1.4–1.6)	1.3 (1.2–1.5)	1.3 (1.1–1.5)	1.3 (1.1–1.6)	1.5 (1.1–2.1)	1.1 (0.7–1.7)
Pooled estimate	1.6 (1.5–1.7)	1.4 (1.4–1.5)	1.3 (1.3–1.4)	1.4 (1.3–1.5)	1.4 (1.3–1.6)	1.3 (1.2–1.5)
*I*^2^	71.5	62.4	26.7	41.2	42.8	44.4

Note: CVD = cardiovascular disease.

**Figure 2:**
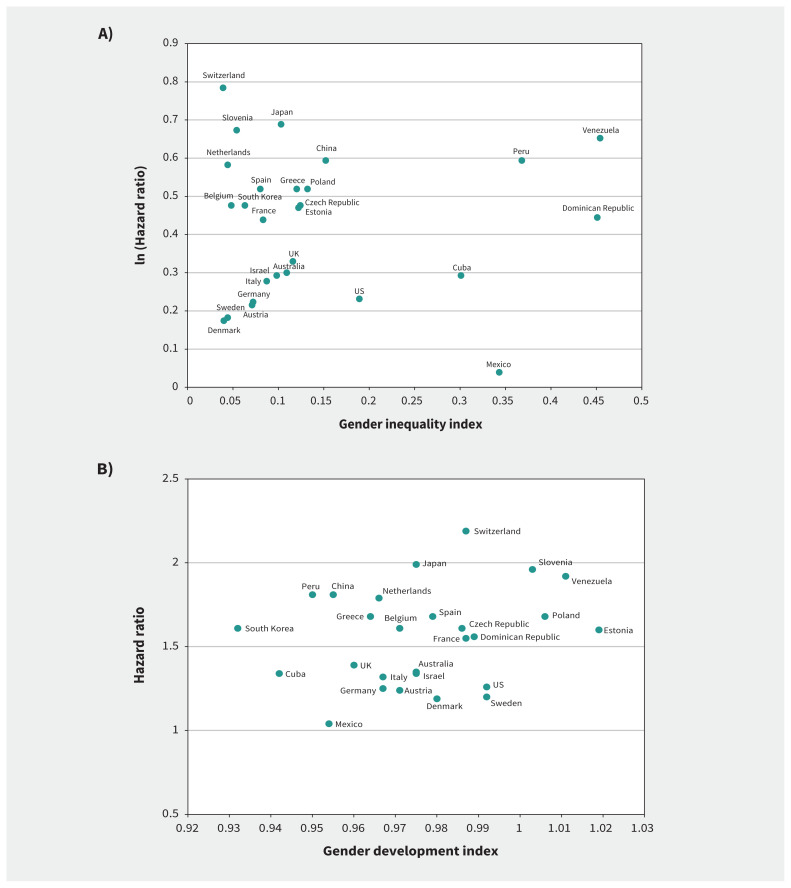
Scatter plots of country-specific hazard ratios for men versus women, adjusted for age, smoking and cardiovascular disease, according to the United Nations’ (A) gender inequality index and (B) gender development index across countries. Gender inequality index: Higher values indicate more gender inequality. Gender development index: 1 indicates good equality of development between women and men. Values < 1 and > 1 suggest less equality of development between women and men.

We observed no clear patterns in the scatter plots between the adjusted hazard ratios and the 2 UN indicators at the country level (Figure 2). Spearman correlation coefficients were 0.05 (*p* = 0.8) for the gender inequality index (Figure 2A) and 0.12 (*p* = 0.5) for the gender development index (Figure 2B). The full models including all factors did not attenuate sex differences in mortality (Appendix 1, Table S3). The sensitivity analyses incorporating time-varying covariates across follow-up waves showed results similar to those of the main analysis (Appendix 1, Table S4). Because birth cohorts had limited effects on the results (Appendix 1, Table S5), the main analyses did not include the birth cohort variable.

## Interpretation

The analysis of a harmonized data set of 12 population-based cohort studies shows that men had a 60% higher mortality risk than women, but this gap varied across countries. Among a wide range of socioeconomic status, lifestyle, health and social factors, only smoking and cardiovascular disease attenuated sex differences in mortality by up to 22%.

The results of this study correspond to the literature on life expectancy and mortality rates^4^,^5^ and highlight sex differences in mortality at older age and substantial heterogeneity across countries. Although the ATHLOS harmonized data set and existing studies mainly used the measure for biological sex, the effects of sex on mortality should include not only physiologic variation between men and women but also the social construct of gender, which differs across societies. In particular, the large variation across countries may imply a greater effect of gender than sex. Although the biology of the sexes is consistent across populations, variation in cultural, societal and historical contexts can lead to different life experiences of men and women and variation in the mortality gap across countries.^6^

Among all factors, smoking had the largest contribution to the difference in mortality between men and women, particularly in the countries where men had more than twice the mortality risk of women (Estonia, Poland and Japan). International studies and primary research in these regions have reported large sex differences in the prevalence of smoking and related morbidity and mortality, with trends that are stable or decreasing in men but increasing in women.^29^–^33^ Although tobacco control polices have been suggested to reduce smoking-related mortality in some Eastern European countries,^32^,^34^ their effects may differ between men and women. We observed differential impacts of smoking and cardiovascular disease on mortality in men and women across countries. This might indicate that male smokers experienced different risk factors or prognosis of chronic conditions than their female counterparts.^35^

The heterogeneity of sex differences in mortality across countries may indicate the substantial impact of gender on healthy aging in addition to biological sex, and the crucial contributions of smoking may also vary across different populations. Public health policies must recognize variation among genders and further incorporate cultural and societal factors within and across countries.^36^ For example, given that opposite trends in prevalence of smoking, associated morbidity and mortality have been observed in men (decreasing) and women (increasing),^32^,^33^,^37^ tobacco control policy-makers should consider changes in gender roles over time and the variation in life experiences across different societies so as to reduce the impact of smoking on the whole population. To strengthen evidence and inform population-level interventions, advanced epidemiologic methods for causal inference are needed to facilitate subgroup and mediation analyses in future research.^38^

### Limitations

Variation in study designs across cohort studies — such as sampling methods, response rates and the length of follow-up periods — might affect representation of older populations in specific countries. In addition, most cohort studies were from high-income countries. This might limit generalizability of the findings. Although all variables were harmonized, methods of data collection — such as mortality data based on family-reported death or national death registry — could still differ across cohort studies, and this may lead to misclassification and variation in measurements. The residual sex differences might be attributed to other factors such as physical activity, but several cohort studies did not have comparable data for harmonization. Data on wealth based on individual or household income could be inaccurate for older participants who were retired. Some cohort studies had more comprehensive data on pension, insurance or other financial information, and we used these to generate the relative wealth quintile where possible. Some lifestyle, health and social factors were likely to change over time, but the results of models incorporating time-varying covariates were similar to the main analysis. Although we obtained the UN gender inequality and development indexes to contextualize the results, these 2 indexes composite several health (life-expectancy) and socioeconomic indicators (education, employment and living standards) and might not sufficiently reflect societal and cultural factors across countries. The change-in-estimate approach might be limited to address potential confounders on different pathways between sex, risk factors and mortality.^38^ However, complex modelling frameworks can be difficult to incorporate into the 2-stage individual participant–data analysis. The results did not indicate to what extent sex differences in mortality could be attributed to either biological sex or gender. For better understanding of gender inequality, it is essential to integrate data on biological, material, behavioural and societal factors over the life course in men and women. Data harmonization may be a fruitful approach for bringing together strengths of existing cohort studies and identifying different mechanisms across genders, populations and generations.^6^

### Conclusion

This study highlights sex inequality in mortality at older age and the crucial contributions of smoking to excess mortality in men. Future research should investigate variation in life experience between men and women and underlying mechanisms across different societies.
